# Insight into the Probiogenomic Potential of *Enterococcus faecium* BGPAS1-3 and Application of a Potent Thermostable Bacteriocin

**DOI:** 10.3390/foods13162637

**Published:** 2024-08-22

**Authors:** Nikola Popović, Katarina Veljović, Dušan Radojević, Emilija Brdarić, Dušan Stevanović, Milica Živković, Milan Kojić

**Affiliations:** 1 Institute of Molecular Genetics and Genetic Engineering, University of Belgrade, Vojvode Stepe 444a, 11042 Belgrade, Serbia; katarinav@imgge.bg.ac.rs (K.V.); dradojevic@imgge.bg.ac.rs (D.R.); emilija@imgge.bg.ac.rs (E.B.); dstevanovic@imgge.bg.ac.rs (D.S.); milicanikolic@imgge.bg.ac.rs (M.Ž.); 2 Department of Research and Development, Institute of Virology, Vaccines, and Sera “Torlak”, Vojvode Stepe 458, 11152 Belgrade, Serbia

**Keywords:** *Enterococcus*, probiogenomics, bacteriocins, *Listeria*, overexpression, preservatives

## Abstract

This study aimed to investigate the probiogenomic features of artisanal bacteriocin-producing *Enterococcus faecium* BGPAS1-3 and the use of the improved pMALc5HisEk expression vector for overexpressing class II bacteriocins and the application of purified bacteriocin 31 in a milk model as a preservative against *L. monocytogenes*. The BGPAS1-3 strain was isolated from traditional fresh soft cheese manufactured in households on a small scale in rural locations surrounding Pale Mountain City in Bosnia and Herzegovina. The whole-genome sequencing approach and bioinformatics analyses revealed that the strain BGPAS1-3 was non-pathogenic to humans. The presence of bacteriocin operons suggested the ability of the isolate to suppress the growth of pathogens. Coding regions for three maturated bacteriocins (bacteriocin 31, bacteriocin 32, and enterocin P) produced by BGPAS1-3 were amplified and expressed in *Escherichia coli* ER2523 using the pMALc5HisEk system. All three bacteriocins were successfully overexpressed and purified after enterokinase cleavage but showed different antimicrobial activity. Bacteriocin 31 showed significantly stronger antimicrobial activity compared with bacteriocin 32. It was the only one that proved to be suitable for use as a food preservative against *L. monocytogenes* in a milk model.

## 1. Introduction

The genus Listeria consists of Gram-positive bacilli that are ubiquitous in various environments and can infect humans or warm-blooded animals through contaminated food [1]. Within this genus, in addition to non-pathogenic species, there are only two pathogenic species for humans and animals, *Listeria monocytogenes* and *Listeria ivanovii* [2]. The *Listeria* outbreaks have a significant economic impact on the food industry [3]. Many cases of listeriosis are linked to outbreaks. At the same time, numerous sporadic cases can also arise from persistent *L. monocytogenes*, underscoring the severe public health and economic consequences of *L. monocytogenes*’ persistence in the food system. This persistence can lead to the contamination of finished products, resulting in numerous recalls, product redesigns, and internal rejections, all of which amplify the economic impact [4]. Moreover, extensive testing programs, which are designed to identify growth niches and environmental persistence, add to the financial burden [5]. Although precisely quantifying the economic impact of *L. monocytogenes* persistence is challenging, it is evident that a significant portion of the overall costs related to *L. monocytogenes* contamination in the food industry stems from the persistent presence of this major foodborne pathogen in processing environments [6]. In addition to that, infections caused by pathogenic species are widespread globally and have significant health importance. Listeriosis is a serious zoonotic and systemic disease affecting human health after the consumption of contaminated foods [7]. *Listeria monocytogenes* is an important foodborne pathogen in humans and a cause of foodborne illness [8]. After being ingested through contaminated food, *L. monocytogenes* infects the intestinal epithelium, passes through the intestinal barrier, and causes systemic infections in other body organs [9]. By producing a soluble cytolysin, listeriolysin O (LLO), and intracellular replication, *L. monocytogenes* leads to a high hospitalization rate and potential mortality in immunocompromised individuals [10]. The ability to produce and form biofilms plays a role in the persistence of *L. monocytogenes* in the environment for long periods [11]. Biofilm formation affects resistance to various antibiotics and organic sanitizers [12]. The biofilm increases cell density, which provides an optimal environment for the exchange of eDNA via conjugation, some of which may code for antibiotic resistance. Cells in biofilms are about 1000 times more resistant to antibiotics than the planktonic form, and one of the reasons is the presence of extracellular polymeric substance (EPS) matrices. Additionally, the EPS matrix protects bacterial cells in deeper layers from antimicrobial agents by limiting diffusion [13,14]. The multiple drug-resistant *L. monocytogenes* strains isolated from environmental and animal sources, as well as various foods, have been extensively reported. These strains are associated with the excessive use of antibiotics in clinical environments and farms [15]. In recent years, the application of alternative approaches in the control of *L. monocytogenes* has been a priority [16,17,18]. Live probiotic strains of lactic acid bacteria (LAB) and their bioactive compounds have become important in controlling *L. monocytogenes* in the food industry [19]. Increased attention has been focused on the ability of LAB to produce bacteriocins, specific proteinaceous substances, which inhibit the growth of pathogens such as *Listeria* and extend the shelf life of food products [20,21]. Many bacteriocins of LAB are effective and safe natural inhibitors of pathogenic and food spoilage bacteria and have been suggested for use as food “preservatives” [22,23]. The enterococci produce bacteriocins, small molecular weight peptides, usually characterized by specific antimicrobial activity against Gram-positive pathogens and spoilage bacteria. Enterococcal bacteriocins or enterocins are classified into four classes: class I, lantibiotic; class II, non-lantibiotic; class III, cyclic enterocin; and class IV, enterocin with a high molecular weight [24]. Most enterocins produced by *Enterococcus* sp. belong to class II and have strong inhibitory effects against *L. monocytogenes*. Class II enterocins are heat-stable, ribosomally synthesized peptides that do not undergo extensive post-translational modifications besides cleavage of a leader peptide during their transport out of the cell [25]. In the food industry, bacteriocins can be applied either as added food additives or through in situ production by starters. The use of bacteriocin-producing enterococci as starter cultures in the production of certain Mediterranean cheeses may prevent spoilage and growth of *L. monocytogenes* [26]. Considering that enterococci can also be pathogenic, using them in the food industry presents challenges [27]. A whole-genome sequencing (WGS)-based method is the only effective method to fully characterize the safety status of *Enterococcus* strains [28] and is in line with the statement of the European Food Safety Authority (EFSA) on the requirements for WGS analysis of strains intended for use in the food industry [29]. Based on whole-genome information, the prediction of potential harmful determinants or genetic features associated with beneficial effects provides a comprehensive insight into potential probiotic strains. Combining the results of WGS and phenotypic tests provides insights into the potential of the selected strain for further application [30]. Although the use of bacteriocins is considered safe [31], in most cases the use of bacteriocins has not been proven to be successful. The reasons for the lower antimicrobial activity are most likely either the degradation or the adsorption of bacteriocins into complex food matrices [32]. The only bacteriocin approved for use in the food industry is Nisin [33]; however, its application is limited due to its low activity in non-acidic environments. Bacteriocins are used in the preservation of various foods, either alone or combination with other conservation methods, most often pasteurization [34].

To our knowledge, this is the first study to report the use of probiogenomics to assess the safety and probiotic properties and use of the improved pMALc5HisEk expression vector for overexpressing class II bacteriocins and application of purified bacteriocin 31 in milk model as a preservative against *L. monocytogenes*.

## 2. Materials and Methods

### 2.1. Bacterial Strains and Growth Conditions

*Enterococcus faecium* BGPAS1-3 from the laboratory collection of the Institute of Molecular Genetics and Genetic Engineering, University of Belgrade, Serbia, was grown in M17 medium (Merck GmbH, Darmstadt, Germany) supplemented with D-glucose (0.5% *w*/*v*) (GM17) at 37 °C. *Escherichia coli* ER2523 (New England Biolabs, Ltd., Hitchin, UK) was grown in Luria Bertani (LB) medium containing 0.5% NaCl, 0.5% yeast extract, and 1% tryptone (Torlak, Belgrade, Serbia) at 37 °C with aeration (180 rpm). Solid medium and soft agar were prepared by adding 1.5% and 0.7% (*w*/*v*) agar (Torlak, Belgrade, Serbia) to the liquid media, respectively. Ampicillin (100 μg/mL) was used for selecting and maintaining transformants. Isopropyl-β-D-1-thiogalactopyranoside (IPTG; Serva, Heidelberg, Germany) was used at suitable concentrations to induce protein expression. For the count of *L. monocytogenes* BG322, IMS1, IMS2, IMS3, and IMS4 strains in the milk model, Difco Oxford Medium Base (Becton, Dickinson and Company, Sparks, MD, USA) was used. Indicator strains and growth conditions are listed in Table 1.

### 2.2. Whole-Genome Sequencing, Standard Analysis, and Data Deposition

The total DNA of *En. faecium* BGPAS1-3 was extracted by the method described previously [35]. Total DNA quality and concentration were evaluated by agarose gel electrophoresis and by a Bio-Spec nano (Shimadzu, Tokyo, Japan) spectrophotometer. The whole-genome sequencing (WGS) of the *En. faecium* BGPAS1-3 genome was performed on the Illumina HiSeq 2500 platform by the MicrobesNG service (MicrobesNG, IMI-School of Biosciences, University of Birmingham, Birmingham, UK) using a 2 × 250 bp paired-end strategy. Library preparation was carried out using the Nextera XT Library Prep Kit (Illumina, San Diego, CA, USA), with minor modifications to the manufacturer’s protocol. Specifically, 2 ng of input DNA was used, and the PCR elongation time was extended to 1 min. The process was executed on a Hamilton Microlab STAR (Hamilton, Reno, NV, USA). Pooled libraries were quantified with a Roche LightCycler 96 qPCR machine (Roche, Indianapolis, IN, USA) using the Kapa Biosystems Library Quantification Kit for Illumina. Trimmomatic v0.30 [36] with a sliding window quality cutoff of Q15 was used for adapter trimming. De novo assembly was performed using SPAdes v3.7 [37], and contigs were annotated using Prokka 1.11 [38]. Kraken software [39] was used to identify the closest available reference genome, and BWA MEM [40] was used for mapping the reads to the reference genome. Draft genome sequences of BGPAS1-3 have been deposited in GenBank under accession number JAUKVL000000000.

### 2.3. Genotypic Characterization for Safety and Probiotic-Related Traits

The *En. faecium* BGPAS1-3 complete genome sequence was analyzed for safety assessment using the online bioinformatics tools accessible at the Center for Genomic Epidemiology: ResFinder 4.1 (https://cge.cbs.dtu.dk/services/ResFinder/; accessed on 26 April 2024). [41] following the thresholds of 60% identity over a length of 60% coverage, respectively. Further, VirulenceFinder (https://cge.food.dtu.dk/services/VirulenceFinder/; accessed on 26 April 2024) [42] and the Virulence Factor of Bacterial Pathogens Database (VFDB) (http://www.mgc.ac.cn/VFs/main.htm; accessed on 26 April 2024) [43] were used to predict potential virulence genes. The identification threshold was set at 90% identity over a length of 60% for VirulenceFinder. Pathogen-Finder v1.1 (http://cge.cbs.dtu.dk/services/PathogenFinder/; accessed on 26 April 2024) [44] was used to investigate acquired pathogenic and antibiotic resistance factors. PlasmidFinder v2.1.1 (http://cge.cbs.dtu.dk/services/PlasmidFinder; accessed on 26 April 2024) [45] was used for plasmid identification against a Gram-positive database with a 60% minimum identity threshold and minimum 60% coverage. For beneficial traits, the BGPAS1-3 complete genome was mined for genes associated with biosynthetic pathways of essential amino acids using the KAAS-KEGG Automatic Annotation Server (http://www.genome.jp/kegg/kaas/; accessed on 26 April 2024) [46] and BAGEL4 (http://bagel4.molgenrug.nl/; accessed on 26 April 2024) [47] for the detection of potential antimicrobial compounds. The Proksee web platform (https://proksee.ca/; accessed on 26 April 2024) was used for circular genome visualization and comparison [48].

### 2.4. DNA Manipulations

For plasmid isolation from *E. coli* ER2523 transformants, a Thermo Fisher Scientific GeneJET Plasmid Miniprep kit was used according to the manufacturer’s instructions (Thermo Scientific, Vilnius, Lithuania). Digestion with restriction enzymes was conducted according to the supplier’s instructions (Thermo Fisher Scientific, Waltham, MA, USA). By the manufacturer’s recommendations, DNA was ligated using T4 DNA ligase (Agilent Technologies, Santa Clara, CA, USA). The standard heat shock transformation method was used for the transformation of *E. coli* with a plasmid [49].

### 2.5. Overexpression of Bacteriocins from Enterococcus faecium BGPAS1-3 in Escherichia coli ER2523

Transformants of *E. coli* ER2523 (pMALc5HisEk_Bac31, pMALc5HisEk_Bac32, and pMALc5HisEk_EntP) were maintained overnight on LB Agar Petri dishes containing ampicillin (100 µg/mL) and glucose (1%) at 30 °C. The next day, new cultures (each 200 mL of LB with 1% glucose and 100 µg/mL ampicillin) were inoculated using a 2% overnight culture and incubated at 30 °C with aeration (180 rpm on a rotatory shaker). Expression of recombinant peptides was induced in the logarithmic growth phase (OD_600_ = 0.8–1.0) with the addition of 0.3 mmol/L (final) IPTG for 3 h. Bacterial cells were collected by centrifugation at 4500× *g* and frozen at −20 °C. Before purification, the level of induction was tested by comparing the amounts of total proteins from the same amount of induced and non-induced cells. Purification including cell lysis, affinity chromatography, and cleavage of the fusion protein with enterokinase was performed according to the manufacturer’s instructions (amylose resin purification, according to pMAL Protein Fusion & Purification System, New England Biolabs, Ltd., UK; Ni-NTA agarose resin purification, according to The QIAexpressionist, Qiagen Gmbh, Hilden, Germany; cleavage with recombinant bovine enterokinase in 1× enterokinase buffer (20 mmol/L Tris-HCl, pH 7.4, 50 mmol/L NaCl, 2 mmol/L CaCl_2_) using 10 U, according to GenScript, Piscataway, NJ, USA), with the addition of a cell lysis step that was performed in column buffer (CB) with 1 mg/mL lysozyme for 30 min on ice. The total protein concentrations were determined using the BSA protein assay kit (Thermo Fisher Scientific, Waltham, MA, USA). Total protein concentrations were measured using the BSA protein assay kit (Thermo Fisher Scientific, Waltham, MA, USA). Proteins from all stages of expression, purification, and proteolysis were analyzed by 12.5% sodium dodecyl sulfate-polyacrylamide gel electrophoresis (SDS-PAGE). For SDS-PAGE, samples were mixed with 2 × sample loading buffer (125 mmol/L Tris-HCl pH 6.8, 10 mmol/L EDTA, 4% SDS, 25% glycerol, 5% β-mercaptoethanol, 0.07% bromophenol blue) at a 1:1 ratio. Before loading onto the gel, samples were denatured by heating at 100 °C for 5 min.

### 2.6. Bacteriocin Activity Assay

To detect bacteriocin activity, an agar-well diffusion assay was conducted [50]. Cultures for the bacteriocin assay were grown without antibiotic selection. A spot-on-the-lawn inhibition assay [51] was used to test the antimicrobial activity of recombinant antimicrobial peptides. The sensitive strain used in bacteriocin tests was *L. monocytogenes* BG322. Inhibition zones were examined after 24 h of incubation at 37 °C, with a clear zone indicating bacteriocin activity. The bacteriocin activity assay was performed in at least two independent experiments. Bacteriocin activity was quantified by performing two-fold serial dilutions of the purified bacteriocins, and activity was expressed in arbitrary units per milliliter (AU/mL). One arbitrary unit (AU) is defined as the reciprocal of the highest dilution of bacteriocin that still produces a clear zone of growth inhibition in the indicator strain [52].

### 2.7. Amplified Fragments and Constructs Sequencing and Sequence Analysis

Amplified fragments and constructs were sequenced by the Macrogen sequencing service (Macrogen Europe, Amsterdam, The Netherlands). Sequence annotation and similarity searches were performed using the BLAST program of the National Center for Biotechnology Information (NCBI) [53]. Open reading frames (ORFs) and restriction enzyme sites were predicted using the DNA Strider3 program.

### 2.8. Effects of Purified Bacteriocin 31 on the Abundance of Listeria monocytogenes in a Milk Model

A milk model was used to investigate the effects of purified bacteriocin 31 on the abundance of *L. monocytogenes* BG322, IMS1, IMS2, IMS3, and IMS4 according to the previously described protocol [54] with slight modifications. Autoclaved reconstituted skimmed milk (11% *w*/*v* RSM) was independently inoculated with 10^7^ CFU/mL of *L. monocytogenes* BG322, IMS1, IMS2, IMS3, and IMS4. Different amounts of BGPAS1-3 purified bacteriocin 31 at final concentrations of 100 AU/mL, 500 AU/mL, and 1000 AU/mL were added. The milk samples were monitored during the 7-day storage at 4 °C. For analysis, serial dilutions of each sample (ranging from 10^−1^ to 10^−7^) were prepared, and 100 μL of each dilution was plated onto Difco Oxford Medium Base agar plates. The plates were then incubated for 48 h at 37 °C under aerobic conditions.

### 2.9. Statistical Analysis

All experiments were independently repeated at least three times, with each experiment performed in triplicate. The data are presented as mean values ± standard deviation across all experiments. For comparisons among multiple groups, two-way ANOVA followed by the Bonferroni post hoc test was applied. Statistical significance was defined as *p* < 0.05. Statistical analyses and graph preparations were carried out using GraphPad Prism 10 software.

## 3. Results

Previous studies have shown that *En. faecium* BGPAS1-3 has a strong direct antilisterial effect, which was observed at a similar level after treatment with heat-killed BGPAS1-3 cells [55]. Furthermore, our previous results show that live and heat-killed BGPAS1-3 cells can prevent tight junction disruption, enable *IL-8* mRNA induction, and stimulate *TGF-β* mRNA expression in differentiated Caco-2 infected with *L. monocytogenes* [55]. Taking into account all these demonstrated features and that the tested strain can synthesize thermostable bacteriocins, in this study we analyzed the genome of BGPAS1-3 using a variety of bioinformatic tools to assess the strain’s safety status and analyzed beneficial features with particular emphasis on the presence of bacteriocins and to clone, overexpress, prove antimicrobial activity, and apply bacteriocins in a milk model for the *L. monocytogenes* control.

### 3.1. Enterococcus faecium BGPAS1-3 Was Predicted as a Non-Human Pathogen

**Figure 1:**
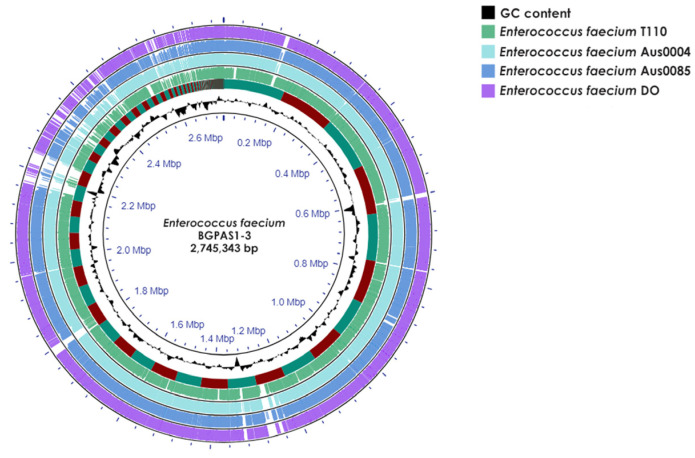
Circular genome map of *Enterococcus faecium* BGPAS1-3 visualized and compared with selected pathogenic (Aus0004, Aus0085, and DO) and probiotic (T110) *Enterococcus faecium* strains using Proksee. Note that the map’s rings are arranged as follows from the inside out: the first ring shows scale marks in megabase pairs (Mbp) and GC content (black); the second ring depicts a comparative analysis of the BGPAS1-3 genome against the T110 genome using BLAST+ 2.12.0 software; and subsequent rings compare the BGPAS1-3 genome with those of strains Aus0004, Aus0085, and DO.

Whole-genome sequencing (WGS) of *Enterococcus faecium* BGPAS1-3 revealed that the genome size was 2,745,343 bp (175 contigs), comprising 2.664 protein-coding sequences (CDS), 66 tRNAs, and 1 rRNA operon, with a guanine-cytosine (GC) content of 37.97% (Figure 1).

Antimicrobial resistance (AMR) analysis was performed using ResFinder (v4.1), which identified two genes involved in resistance to antibiotics. These two genes corresponded to a homolog of *aac(6′)-Ii* involved in aminoglycoside resistance (% identity, 99.82; query/HSP length, 549/549; accession number, L12710) and a homolog to *msr(C)* involved in MLS (Macrolide, Lincosamide and Streptogramin B) (% identity, 98.98; query/HSP length, 1479/1479; accession number, AY004350) (Supplementary Table S1).

Virulence factors screening was performed using VirulenceFinder (v2.0). The analysis discovered the presence of two virulence genes: *acm* (98.98%; 1866/2166; CP003351.1) and *efaA^fm^* (90.71%; 861/879; AF042288.1), which encode collagen-binding protein and cell wall adhesin, respectively. Moreover, the Virulence Factor of Bacterial Pathogens Database (VFDB) was used to search for virulence factors and showed that the BGPAS1-3 genome contained genes responsible for adhesion (*ebpA*, *ebpB*, *ebpC*, *srtC*, and *efaA*), antiphagocytosis (*cpsA*/*uppS*, *cpsB*/*cdsA*, *cpsC*, and *cpsK*), biofilm formation (*bopD*), and immune evasion (*cps4I*) (Supplementary Table S2).

The pathogenic potential of BGPAS1-3 was determined via PathogenFinder (v1.1.). Based on the results, BGPAS1-3 was predicted as a non-human pathogen (the probability of being a human pathogen was 0.262), with five not-pathogenic and two pathogenic families. PlasmidFinder (accessed on 26 April 2024) was used for plasmid identification against a Gram-positive database with a 60% minimum identity threshold and minimum 60% coverage. Seven plasmid sequences were discovered: rep18a (99.89%), rep29 (100%), repUS42 (87.29%), repUS53 (88.8%), repUS15 (99.52%), rep2 (99.93%), and rep1 (96.97%) (Supplementary Table S3).

### 3.2. Enterococcus faecium BGPAS1-3 Contains Genes Predicted to Be Involved in Biosynthetic Pathways of Essential Amino Acids

Desirable traits of the probiotic strains included the production of essential amino acids. Using the KAAS-KEGG Automatic Annotation Server to search the BGPAS1-3 genome, genes involved in biosynthetic pathways of essential amino acids were found. It was noticed that the BGPAS1-3 genome contained genes involved in the biosynthesis of the essential amino acids alanine, aspartate, arginine, cysteine, and valine (Supplementary Table S4).

### 3.3. Enterococcus faecium BGPAS1-3 Contains Operons/Gene Clusters Predicted to Encode the Production of Four Antimicrobial Peptides

The genome of *En. faecium* BGPAS1-3 was analyzed for genes encoding bacteriocins and non-bactericidal post-translationally modified peptides using the BAGEL4 online tool. The BAGEL4 analysis identified four candidate genes in *En. faecium* BGPAS1-3 that are associated with the production of antimicrobial peptides: enterolysin A, bacteriocin 31, bacteriocin 32, and enterocin P. Based on the analysis in BAGEL4, enterolysin A showed a similarity of 36.4% to those previously expressed where activity was obtained and was not the subject of further investigations (Table 2, Supplementary Figure S1).

### 3.4. Overexpression of Bacteriocin Genes from Enterococcus faecium BGPAS1-3 in Escherichia coli ER2523

**Figure 2:**
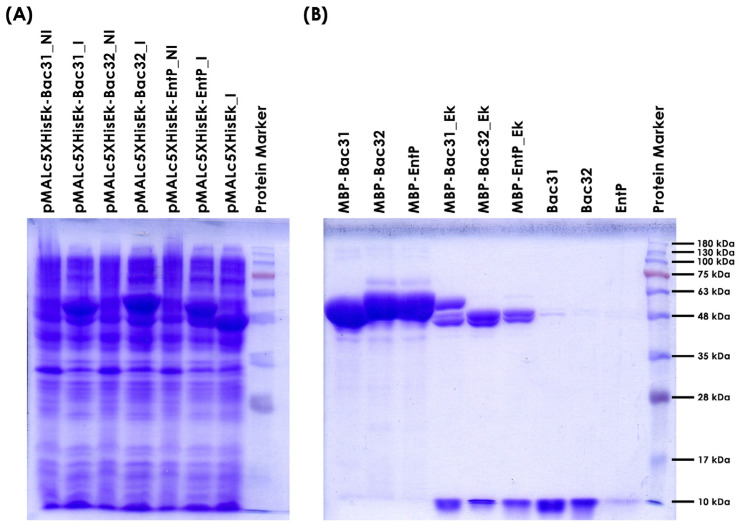
SDS-PAGE analysis of three *Enterococcus faecium* BGPAS1-3 bacteriocins expression and purification from *E. coli* ER2523. (**A**) Analysis of the level of induction/expression of three enterococcal bacteriocins (tag MBP-bacteriocin hybrid protein) in *E. coli* ER2523. Total proteins of *E. coli* ER2523 carrying pMALc5HisEk_Bac31, pMALc5HisEk_Bac32, pMALc5HisEk_EntP, and empty vector pMALc5HisEk before (NI) and after induction (I) with 0.3 mmol/L of IPTG were loaded on 12.5% SDS PAGE gel. (**B**) Analysis of the level of tag-bacteriocin purification (MBP-), efficiency of enterokinase digestion (Ek), and tag release of three enterococcal bacteriocins on 15% SDS PAGE gel.

To test which of the thermostable bacteriocins produced by *En. faecium* BGPAS1-3 are naturally active on *L. monocytogenes* BG322, three bacteriocin genes (bacteriocin 31, bacteriocin 32, and enterocin P) were amplified by PCR using primers listed in Table 3. Amplified fragments of the expected sizes were purified, digested with corresponding restriction enzymes (bacteriocin 31 with *Bam*HI, and bacteriocin 32 and enterocin P with *Hin*dIII), and ligated into pMALc5HisEk [56] predigested with *Bsa*BI-*Hin*dIII or *Bsa*BI-*Bam*HI. The ligation mixtures were transformed into ER2523 (New England Biolabs) competent cells. Ampicillin-resistant transformants were screened for the presence of corresponding fragments by plasmid isolation and restriction enzyme (*Sac*I-*Hin*dIII) analysis. Promising constructs (three per cloning) were sequenced using the pMalE primer to confirm the presence of the corresponding DNA fragment in frame with the maltose binding protein gene. Two clones per bacteriocin were stored in LB with 15% glycerol at −80 °C until the overexpression experiments were performed (Figure 2).

Furthermore, the sequences of the expressed enterocin P and bacteriocin 32 proteins from strain BGPAS1-3 were 100% identical to those previously expressed where activity was obtained. Bacteriocin 31 showed the greatest difference, characterized by changes in three amino acids (Supplementary Figure S2).

### 3.5. Purified Bacteriocins Are Active against Listeria sp. and Lactic Acid Bacteria

**Figure 3:**
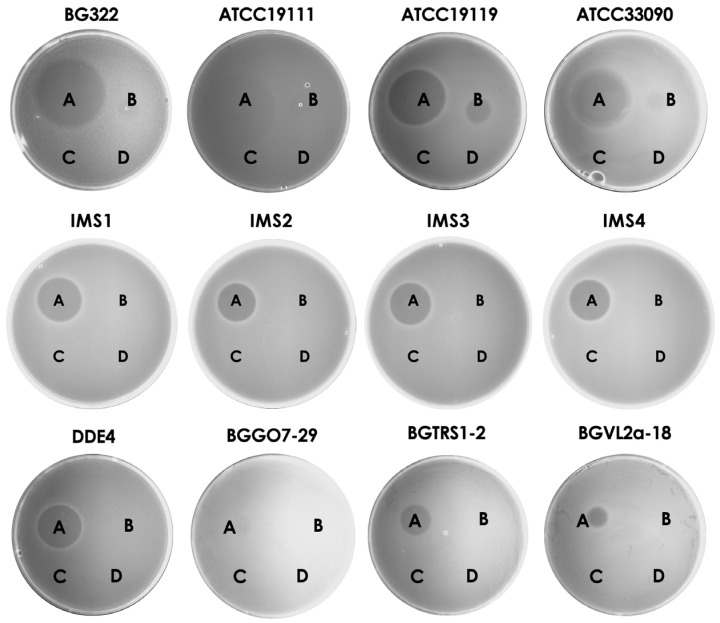
Antimicrobial effect of purified bacteriocins on indicator strains. Note: A—bacteriocin 31; B—bacteriocin 32; C—enterocin P; D—Enterokinase buffer.

After cloning and purification of three thermostable bacteriocins, the antimicrobial potential against different species was tested. It was shown that the purified bacteriocin 31 and bacteriocin 32 were active against *L. monocytogenes* ATCC19111, *L. monocytogenes* BG322, *L. monocytogenes* IMS1, *L. monocytogenes* IMS2, *L. monocytogenes* IMS3, *L. monocytogenes* IMS4, *L. ivanovii* ATCC19119, and *L. inocua* ATCC33090, while bacteriocin 31 exhibited much larger zones of inhibition and showed additional antimicrobial activity against *En. faecium* DDE4, *Lactobacillus plantarum* BGGO7-29, *Lb. plantarum* BGVL2a-18, and *Leuconostoc mesenteroides* subsp. *cremoris* BGTRS1-2. Enterocin P did not show antimicrobial activity against any of the tested indicator strains (Figure 3).

**Figure 4:**
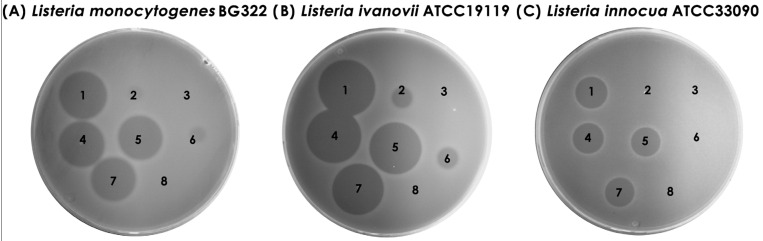
Synergistic effect of purified bacteriocins. Note: 1—bacteriocin 31; 2—bacteriocin 32; 3—enterocin P; 4—the same volume of bacteriocin 31 and bacteriocin 32; 5—the same volume of bacteriocin 31 and enterocin P; 6—the same volume of bacteriocin 32 and enterocin P; 7—the same volume of bacteriocin 31, bacteriocin 32, and enterocin P; 8—enterokinase buffer.

Further, the same volume used for testing individual bacteriocins was applied in combination to investigate the synergistic effect of purified bacteriocins. It was demonstrated that bacteriocins did not exhibit a synergistic effect, and the size of the antimicrobial zone on the indicator strain was the same in bacteriocin combination, as well as after the application of bacteriocin 31 alone (Figure 4).

**Figure 5:**
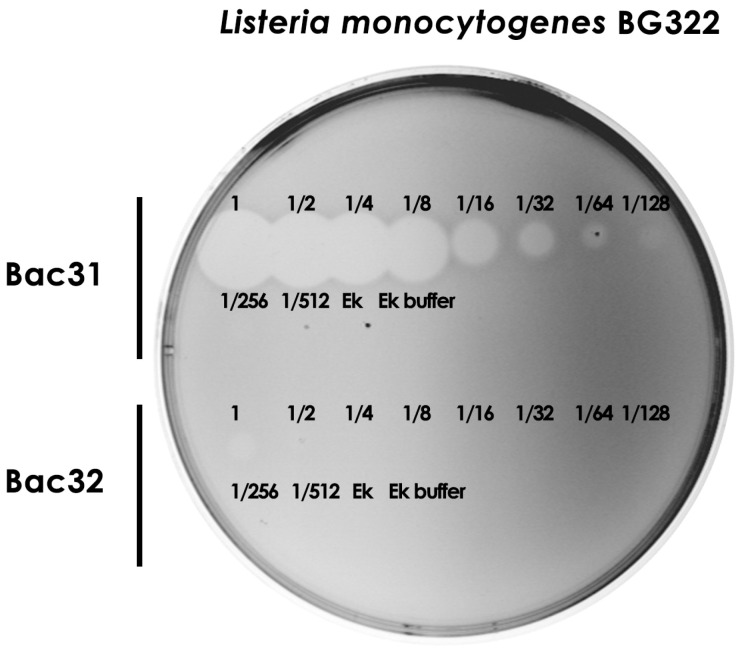
Determination of arbitrary units per milliliter of purified bacteriocins. Note: Fractions represent a two-fold dilution of purified bacteriocin in Ek buffer. Ek—enterokinase; Ek buffer—enterokinase buffer; Bac31—bacteriocin 31; Bac32—bacteriocin 32.

Since we demonstrated the strong antimicrobial activity of bacteriocin 31 and the weak antimicrobial activity of bacteriocin 32, the next goal was to determine arbitrary units per milliliter/mg of purified bacteriocins. We showed that bacteriocin 31 retained its antimicrobial activity after the eight two-fold dilutions against BG322, while bacteriocin 32 lost its activity after the second two-fold dilution. Since the concentrations of both bacteriocins after purification were similar (bacteriocin 31, 2.3 ± 0.3 μg/μL, and bacteriocin 32, 2.8 ± 0.2 μg/μL), it was concluded that bacteriocin 31 was more potent against all *L. monocytogenes* strains (Figure 5).

### 3.6. Application of Purified Bacteriocin 31 Effectively Reduces Listeria monocytogenes in a Milk Model

**Figure 6:**
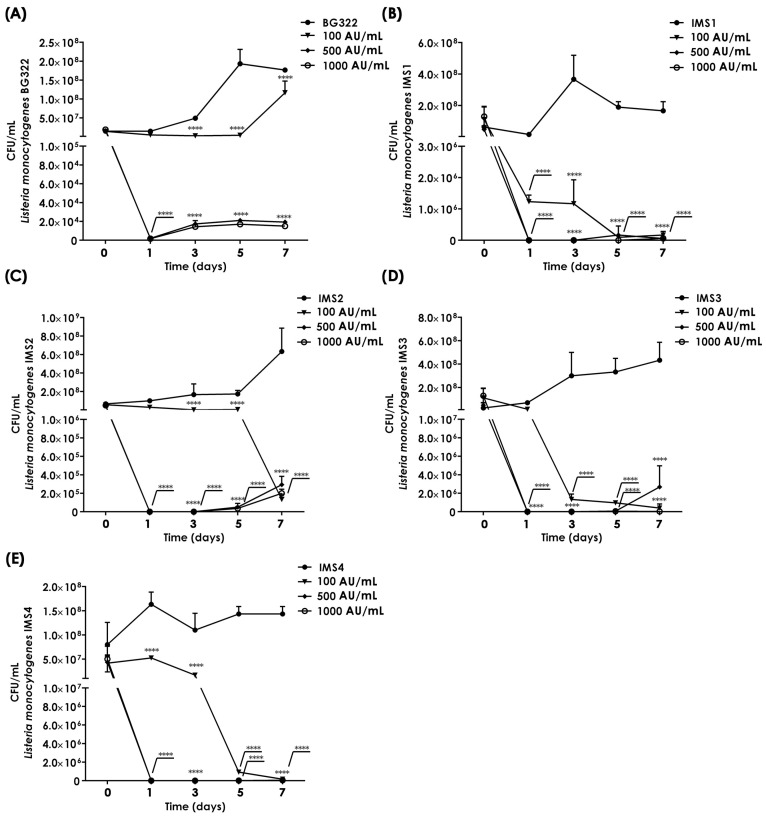
Reduction in *Listeria monocytogenes* in a milk model with purified bacteriocin 31. *L. monocytogenes* BG322 (**A**), *L. monocytogenes* IMS1 (**B**), *L. monocytogenes* IMS2 (**C**), *L. monocytogenes* IMS3 (**D**), and *L. monocytogenes* IMS4 (**E**). Note: Statistical significance *p* < 0.05 ****.

Although the application of bacteriocin and/or bacteriocin-producing starters is considered a good way to protect a food product from pathogenic and food spoilage bacteria, the limiting factor is the weak activity of bacteriocins in complex food systems or the sensitivity of bacteriocins to various technological procedures [57]. Our study demonstrated that purified bacteriocin 31 (100, 500, and 1000 AU/mL) had a significant inhibitory effect after three days of storage on the growth of the *L. monocytogenes* BG322, *L. monocytogenes* IMS1, *L. monocytogenes* IMS2, *L. monocytogenes* IMS3, and *L. monocytogenes* IMS4 in the milk model. A decrease in the number of *L. monocytogenes* BG322 during three days of storage by applying different concentrations of the purified bacteriocin (100, 500, and 1000 AU/mL) was observed from 1.4 × 10^7^ CFU/mL to 5 × 10^6^ CFU/mL, 2.1 × 10^3^ CFU/mL, and 1.6 × 10^3^ CFU/mL, respectively. Also, during the additional four days of storage, the number of *L. monocytogenes* BG322 compared with the control milk was reduced, and the application of the highest concentration of bacteriocin 31 failed to eliminate *L. monocytogenes* BG322 from the milk in 5 days (Figure 6A). Similarly, the application of bacteriocin 31 in three concentrations reduced the presence of *L. monocytogenes* isolated from different types of cheese. The lowest applied concentration of bacteriocin 31 reduced the abundance of all four *L. monocytogenes* after the third day of storage, while concentrations of 500 and 1000 AU/mL reduced the abundance from about 10^8^ to 27–57 CFU/mL. After the fifth and seventh days of storage, with the application of the highest concentration of 1000 AU/mL, the abundance of all four *L. monocytogenes* was significantly reduced compared with untreated milk samples. On the seventh day, the number of *L. monocytogenes* IMS1 compared with the control milk was reduced with the highest concentration from 2.67 × 10^8^ to 6.00 × 10^4^ CFU/mL (Figure 6B), *L. monocytogenes* IMS2 from 6.33 × 10^8^ to 2.93 × 10^5^ CFU/mL (Figure 6C), *L. monocytogenes* IMS3 from 4.33 × 10^8^ to 2.67 × 10^4^ CFU/mL (Figure 6D), and *L. monocytogenes* IMS4 from 1.43 × 10^8^ to 5.00 × 10^4^ CFU/mL (Figure 6E).

## 4. Discussion

Nowadays, there is a tendency to consume ready-to-eat food without or with minimal food processing to reduce the nutrients lost [58]. On the other hand, eating ready-to-eat food might contaminate it with pathogens, which can cause illnesses in humans. One of the important foodborne pathogens is *L. monocytogenes*, which causes listeriosis. Listeriosis is a very serious foodborne disease among high-risk population groups such as the immunocompromised, which is associated with a high mortality rate [59]. Additionally, the consumption of raw milk and raw milk products or post-pasteurization contamination can cause listeriosis outbreaks. There has been a growing need for alternative, natural agents for food preservation and extending food’s shelf life in recent years. Bacteriocins represent safe and harmless alternatives in the control of pathogenic bacteria in food [60]. Because of bacteriocin production, many LABs have been successfully applied to inhibit foodborne pathogens [61,62]. Among LAB species, *Enterococcus* sp. is generally used as a nonstarter lactic acid bacteria (NSLAB) in a variety of cheeses, especially artisan cheeses produced in southern Europe [63]. *Enterococcus* species do not have a generally recognized as safe (GRAS) status, despite the knowledge that the pathogenesis of enterococci is a strain-specific property and is more common to clinical enterococci than to food-based enterococci [64]. Due to their controversial nature, varying from pathogens to probiotics [65], one of the aims of this work was to apply a WGS-based approach for safety assessment and probiotic potential analysis of bacteriocin-producing *En. faecium* BGPAS1-3 originating from young cheese. The genome size of enterococci varies between 2.7 and 3.6 Mb, with a GC content ranging from 37% to 45%. According to Dublin and Palmer [66], the commensal enterococcal strains have smaller genomes compared with the clinical isolates, where genomic variability is caused by the presence of foreign genetic material. The BGPAS1-3 genome size is 2.75 Mb with a total of 2664 protein-coding genes and contains seven plasmids, and when compared with the pathogenic Aus0004, Aus0085, and DO, as well as the probiotic strain T110, differences in sequences are observed. Similar to our study, a comparison of the genomes of probiotic and pathogenic enterococci revealed that the probiotic strain *Enterococcus faecalis* EF-2001 is significantly different from the pathogenic strains [67]. Analysis of the draft genome using bioinformatics programs showed that *En. faecium* BGPAS1-3 is not pathogenic. However, BGPAS1-3 carries the enterococcal intrinsic resistance gene *msr(C)*, responsible for resistance to macrolides and *aac(6′)-Ii*, which encodes an aminoglycoside acetyltransferase responsible for low-level resistance to aminoglycoside. The previous study showed that BGPAS1-3 was sensitive to erythromycin and resistant to low-level gentamicin [65]. In addition, it was found that BGPAS1-3 did not harbor any AMR genes reported for pathogenic enterococci, such as vancomycin resistance genes [68,69]. Moreover, the identification of virulence genes is crucial before classifying enterococci strains as a probiotic. After screening for the presence of virulence genes, genes associated with adhesion, biofilm formation, and antiphagocytosis were found in the BGPAS1-3 genome. In previous studies, a correlation between the presence of these genes and colonization ability was shown. It is well known that adhesion is essential for commensal and probiotic enterococci to promote colonization, persist, and avoid elimination from the host [65]. For beneficial properties, the BGPAS1-3 genome was mined for genes associated with essential amino acid biosynthetic pathways. It has been shown that the BGPAS1-3 genome contains genes involved in the biosynthesis of five amino acids: alanine, aspartate, arginine, cysteine, and valine. A previous study showed that *En. faecium* 17OM39 has the potential to synthesize essential amino acids, and Ghattargi and coauthors [70] assumed that these amino acids serve as precursors for the synthesis of short-chain fatty acids. *Enterococcus* sp. produces a wide diversity of bacteriocins, called enterocins, ribosomally synthesized antimicrobial peptides with specific antimicrobial activity to inhibit the growth of species closely related to the producing bacteria [71]. They have been widely studied because of their activities against some foodborne pathogens and spoilage bacteria [20]. Most enterocins are class II bacteriocins and are defined as unmodified and heat stable [24]. One of the goals of this study was the cloning and overexpression of the thermostable bacteriocins of the BGPAS1-3 strain, which was shown in a previous study to contribute to the reduction in *L. monocytogenes* ATCC19111 adhesion to epithelial cells [55,72]. Using the BAGEL4 software, genes for four bacteriocins (enterolysin A, bacteriocin 31, bacteriocin 32, and enterocin P) were found in the BGPAS1-3 genome. Enterocin P and bacteriocin 31 are classified as “Class II.1. enterocins of the pediocin family” according to Franz and co-authors [17], whereas bacteriocin 32 is classified as “Class II.3. other linear nonpediocin-like enterocins”. Furthermore, three small thermostable bacteriocins (bacteriocin 31, bacteriocin 32, and enterocin P) of BGPAS1-3 were selected for cloning and overexpression. All three bacteriocins have been previously described [24], but bacteriocins from strain BGPAS1-3 showed amino acid differences, especially in the leader peptide sequence. Our previous studies demonstrated that the enhanced *E. coli* pMAL expression system was highly effective for expressing lactococci-like peptides [56,73]. Consequently, we successfully used this system to express and purify three bacteriocins from strain BGPAS1-3. It was surprising to observe a significant variation in the antimicrobial activity of the bacteriocins expressed in *E. coli*: bacteriocin 31 exhibited exceptional specific antimicrobial activity, bacteriocin 32 displayed weak activity, and enterocin P showed no detectable activity. Similar results were obtained with expressed potential bacteriocins of *L. lactis* S50 [73]. For strain BGPAS1-3, the antimicrobial peptides responsible for its antimicrobial activity have not been previously purified or characterized. Therefore, it is unclear whether the analyzed bacteriocins exhibit antimicrobial activity in the natural strain or if the expression system in *E. coli* fails to produce the active form of these peptides. Further research will focus on determining whether additional processing is required for these bacteriocins’ proper synthesis and activity. Enterococcal bacteriocins have been successfully expressed in various heterologous systems, including *E. coli* [74]. Interestingly, no antimicrobial activity was obtained in this study, although the sequence of the expressed enterocin P protein from strain BGPAS1-3 was 100% identical to the previously expressed one where activity was obtained. The difference is that in the previous study, together with enterocin P, the authors expressed a potential protein responsible for immunity [75]. Bacteriocin 31, which showed the greatest difference in the amino acid sequence, showed the highest antimicrobial activity, but considering that we do not have both clones (bacteriocin 31 from BGPAS1-3 and other enterococci), we cannot make a comparison and claim whether it is a consequence of changed amino acids or all clones can exhibit high antimicrobial activity. Site-specific mutagenesis of the pMAL5cHisEk_Bac31 clone, which is planned in the future, will provide an answer about the contribution of individual amino acids to the antimicrobial activity and spectrum of action.

This study showed that purified bacteriocins (bacteriocin 31 and bacteriocin 32) had antimicrobial activities against bacteria from the genera *Listeria*, *Enterococcus*, *Lactobacillus*, and *Leuconostoc*. While previous studies have shown that enterocin P is active against LAB and foodborne pathogens like *L. monocytogenes* [76], this study found no evidence of activity for enterocin P. One of the approaches to eliminating foodborne pathogens is a combination of bacteriocins that show a synergistic effect. The effect of Nisin with other antimicrobial compounds in the effective elimination of pathogens is known [34]. Therefore, we examined the synergistic effect of three bacteriocins in reducing the number of *L. monocytogenes*, and we showed that the use of bacteriocin 31, bacteriocin 32, and enterocin P does not have a synergistic effect. However, it remains an open question for future studies of the synergistic effect of these bacteriocins with technological processes of food production. Bearing in mind that the examined bacteriocins are thermostable, the effect of synergism should be examined with processes that require high temperatures. The application of bacteriocins in milk and the subsequent heat treatment of milk are possible means to eliminate *L. monocytogenes*. In this study, we showed that the application of the most potent purified bacteriocin 31 at different concentrations was able to reduce *L. monocytogenes* BG322, IMS1, IMS2, IMS3, and IMS4 strains in the milk model after three days of storage. However, during storage at 4 °C, even the highest concentration of bacteriocin failed to eliminate *L. monocytogenes*. Dal Bello and coauthors proposed the pretreatment of milk before cheese production as the most effective method in the biocontrol of *L. monocytogenes* [77].

## 5. Conclusions

Probiogenomic analysis of *En. faecium* BGPAS1-3 allows us to better understand its safety status and probiotic potential. Also, these data helped us to investigate the potential application of live strains and holobiotics in the food industry. However, due to controlled application and efficiency, the purified thermostable bacteriocins provide a more practical solution for combating foodborne pathogens. The use of practical tools for the overexpression of such bacteriocins contributed to an efficient reduction in *L. monocytogenes* in milk. In this study, we demonstrated that some enterococcal bacteriocins could be successfully expressed in massive amounts in *E. coli* hosts and applied in food preservation. However, additional experiments are necessary to establish a correlation between the antimicrobial potential, the spectrum of activity, and the amino acid sequence of the bacteriocins.

## Figures and Tables

**Table 1:** Indicator strains used in this study.

Bacterial Strain	Growth Conditions	Source
*Listeria monocytogenes* ATCC19111	GM17; 37 °C	ATCC ^a^
*Listeria monocytogenes* BG322	GM17; 37 °C	Laboratory collection
*Listeria monocytogenes* IMS1	GM17; 37 °C	Laboratory collection
*Listeria monocytogenes* IMS2	GM17; 37 °C	Laboratory collection
*Listeria monocytogenes* IMS3	GM17; 37 °C	Laboratory collection
*Listeria monocytogenes* IMS4	GM17; 37 °C	Laboratory collection
*Listeria ivanovii* ATCC19119	GM17; 37 °C	ATCC
*Listeria inocua* ATCC33090	GM17; 37 °C	ATCC
*Enterococcus faecium* DDE4	GM17; 37 °C	Laboratory collection
*Strepotococcus thermophiles* BGKMJ1-36	GM17; 37 °C; CO_2_	Laboratory collection
*Lactobacillus delbrueckii* subsp. *bulgaricus* BGVL1-21	MRS ^b^; 37 °C; CO_2_	Laboratory collection
*Lactococcus lactis* subsp. *lactis* BGMN1-596	GM17; 30 °C	Laboratory collection
*Lactobacillusplantarum* BGGO7-29	MRS; 37 °C; CO_2_	Laboratory collection
*Leuconostocmesenteroides* subsp. *cremoris* BGTRS1-2	GM17; 30 °C	Laboratory collection
*Lactobacillusplantarum*BGVL2a-18	MRS; 37 °C; CO_2_	Laboratory collection
*Lactococcuslactis* subsp. *lactis* BGVL2-8	GM17; 30 °C	Laboratory collection
*Lactococcuslactis* subsp. *lactis* BGTRK4-21	GM17; 30 °C	Laboratory collection
*Lactococcus lactis *subsp. *lactis* biovar. diacetylactis BGTRK10-2	GM17; 30 °C	Laboratory collection
*Lactococcuslactis* subsp. *cremoris* BGTRM1-22	GM17; 30 °C	Laboratory collection

Notes: ^a^ ATCC-American Type Culture Collection, Manassas, VA, USA; ^b^ MRS de Man, Rogosa and Sharpe (Merck, GmbH, Darmstadt, Germany).

**Table 2:** Results of BAGEL4 search of *Enterococcus faecium* BGPAS1-3 genome sequence.

Bacteriocin Name	Amino Acid Sequence	The Node of the Genome *Enterococcus faecium* BGPAS1-3	The Best Score for Identity
Bacteriocin 31	MKKKFVSIFMILGIVLLSVSTLGITVDAATYYGNGVYCNTQKCWVDWNKASKEIGKIIVNGWVQHGPWAPR	56	98% class II bacteriocin [*Enterococcus*] ID: WP_048340632.1
Bacteriocin 32	MKKTKLLVASLCLFSSLLAFTPSVSFSQNGGVVEAAAQRGYIYKKYPKGAKVPNKVKMLVNIRGKQTMRT CYLMSWTASSRTAKYYYYI	44	100% MULTISPECIES: hypothetical protein [Bacteria] ID: WP_002313303.1
Enterocin P	MTNFGTKVDAATRSYDNGIYCNNSKCWVNWGEAKENIAGIVISGWASGLAGMGH	49	Class II bacteriocin [*Enterococcus*] ID: WP_002298900.1

Note: Underlined amino acid sequences indicate possible leader sequences according to [24].

**Table 3:** List of primers used in this study.

Primer Name	Sequence 5′-3′	Source/ Reference
EntP_Fw	AGCTACGCGTTCATATGATAATGG	This study
EntP_Rev	TAG**AAGCTT**AATGTCCCATACCTGCCAAGCCAGAAGCCC	This study
Bac31_Fw	AGCAACTTATTATGGAAATGGTG	This study
Bac31_Rev	AAC**GGATCC**TTTCTATCTAGGAGCCC	This study
Bac32_Fw	ATTCACCCCTTCTGTTTCATTTTCTC	This study
Bac32_Rev	TTT**AAGCTT**ACTAAATGTAGTAATAATATTTGGC	This study

Note: The recognition sequences for *Hin*dIII (**AAGCTT**) and *Bam*HI (**GGATCC**) restriction enzymes (in reverse primers) are underlined and bolded.

## Data Availability

The data presented in this study are available on request from the corresponding authors. Draft genome sequences of BGPAS1-3 have been deposited in GenBank under accession number JAUKVL000000000. https://www.ncbi.nlm.nih.gov/nuccore/JAUKVL000000000 (accessed on 13 July 2023).

## Supplementary Materials

The following supporting information can be downloaded at https://www.mdpi.com/article/10.3390/foods13162637/s1: Supplementary Figure S1: The presence of genes encoding bacteriocins and non-bactericidal post-translationally modified peptides using the BAGEL4 online tool; Supplementary Figure S2: Comparison of amino acid sequences of *Enterococcus faecium* BGPAS1-3 bacteriocin 31 and representative bacteriocin 31; Supplementary Table S1: Putative antimicrobial resistance genes in *Enterococcus faecium* BGPAS1-3; Supplementary Table S2: Putative virulence factors in *Enterococcus faecium* BGPAS1-3 and comparison with other *Enterococcus faecium* strains; Supplementary Table S3: Putative plasmids identification against a Gram-positive database using PlasmidFinder of *Enterococcus faecium* BGPAS1-3; Supplementary Table S4: Putative genes involved in the synthesis of essential amino acids in *Enterococcus faecium* BGPAS1-3.
