# Supplementary material for: Insight into the Probiogenomic Potential of Enterococcus faecium BGPAS1-3 and Application of a Potent Thermostable Bacteriocin
Source: Foods. 2024 Aug 22;13(16):2637. doi: 10.3390/foods13162637 (PMC11353538; doi:10.3390/foods13162637)
Supplement: Supplementary file 1 [file foods-13-02637-s001.zip › Supplementary Figures.pdf]

Supplementary Figure S1. The presence of genes encoding bacteriocins and non-bactericidal post-translationally modified peptides using the BAGEL4 online tool.

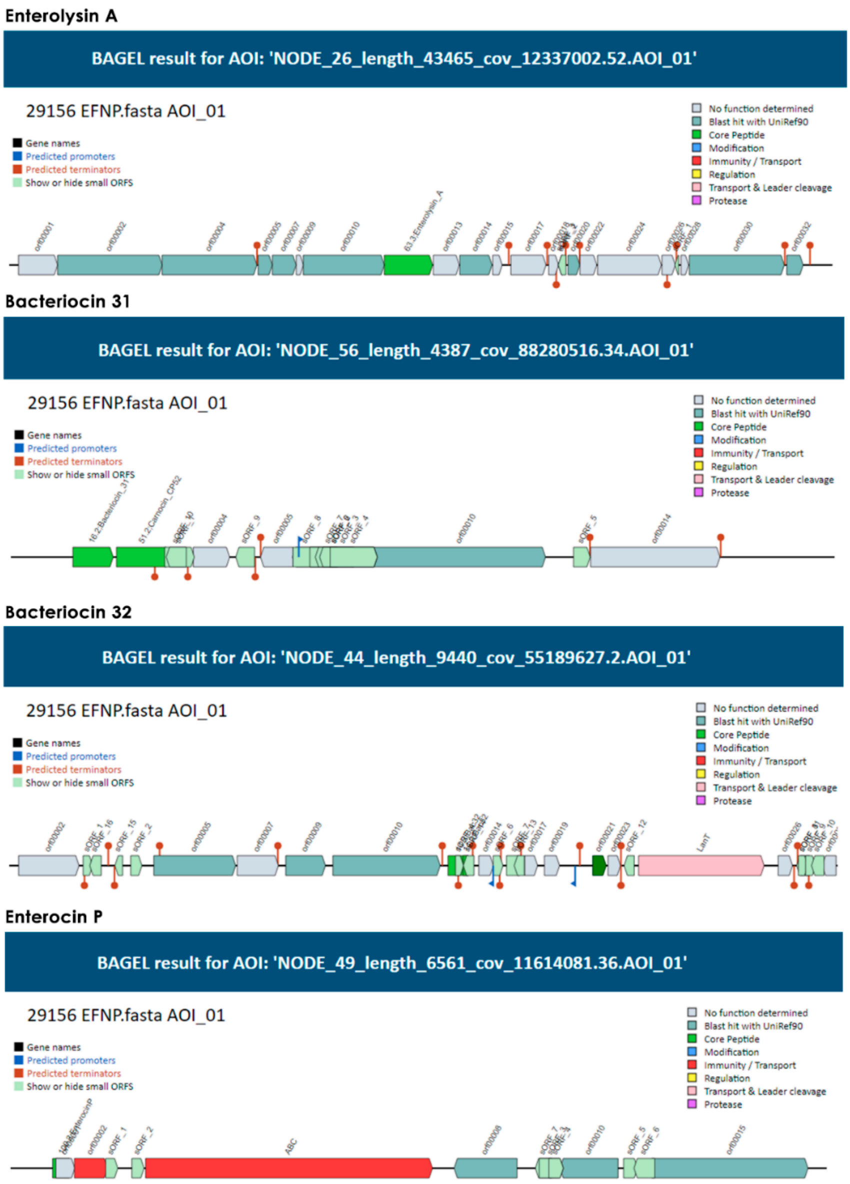

Supplementary Figure S2. Comparison of amino acid sequences of *Enterococcus faecium* BGPAS1-3 bacteriocin 31 and representative bacteriocin 31.
Note: Identical residues are in bold in black. The differences between the two bacteriocins are represented in green (V-L and K-R) and red (T-K).
Note: V - valine; L - leucine; K - lysine; R-arginine; T – threonine

```
                                10        20        40        40
Bac31-BGPAS1-3      1 ATYYGNGVYCNTQKCWVDWNKASKEIGKIIVNGWVQHGPWAPR 43
                      ATYYGNG+YCN QKCWVDWNKAS+EIGKIIVNGWVQHGPWAPR =
Bacteriocin 31.     1 ATYYGNGLYCNKQKCWVDWNKASREIGKIIVNGWVQHGPWAPR 43
                                10        20        30        40
```

Identity = 93.023% (40/43); Homology = 4.651% (2/43) Total = 97.674% (42/43)
