# Supplementary material for: Insight into the Probiogenomic Potential of Enterococcus faecium BGPAS1-3 and Application of a Potent Thermostable Bacteriocin
Source: Foods. 2024 Aug 22;13(16):2637. doi: 10.3390/foods13162637 (PMC11353538; doi:10.3390/foods13162637)
Supplement: Supplementary file 1 [file foods-13-02637-s001.zip › Supplementary Tables.pdf]

Supplementary Table S1. Putative antimicrobial resistance genes in *Enterococcus faecium* BGPAS1-3.

Antibiotic	Gene	Alignment Length/Gene Length	Contig	Position in contig	Identity	Accession number
Macrolides	*msrC*	1479/1479	NODE_8_length_99210_cov_14.370619	16924..18402	98.98	AY004350
aminoglycosides	*aac(6')-Ii*	549/549	NODE_1_length_176348_cov_12.114538	100858..101406	99.81	L12710
Heat	*clpL*	2115/2115	NODE_32_length_22852_cov_11.273223	11495..13609	99.24	CP023753

Supplementary Table S2. Putative virulence factors in *Enterococcus faecium* BGPAS1-3 and comparison with other *Enterococcus faecium* strains.

**VFclass**	**Virulence factors**	**Related genes**	**BGPAS1-3 (Prediction) draft (draft)**	***E.faecium* Aus0004 chromosome (NC_017022)**	***E.faecium* Aus0085 chromosome (NC_021994)**	***E.faecium* DO chromosome (NC_017960)**	***E.faecium* NRRL B-2354 chromosome (NC_020207)**
Adherence	AS	Undetermined	-	-	-	-	-
		Undetermined	-	-	-	-	-
		asa1	-	-	-	-	-
		prgB/asc10	-	-	-	-	-
	Ace	*ace*	-	-	-	-	-
	Acm	*acm*	-	EFAU004_02292	EFAU085_02356	HMPREF0351_12281	M7W_2305
	Ebp pili	*ebpA*	orf02541	EFAU004_00930	EFAU085_01416	HMPREF0351_11394	-
		ebpB	orf02540	EFAU004_00931	EFAU085_01415	HMPREF0351_11393	-
		ebpC	orf02539	EFAU004_00932	EFAU085_01414	HMPREF0351_11392	-
		srtC	orf02538	EFAU004_00933	EFAU085_01413	HMPREF0351_11391	-
	EcbA	*ecbA*	-	EFAU004_01867	EFAU085_01897	HMPREF0351_11828	-
	EfaA	*efaA*	orf02045; orf02434	EFAU004_00491	EFAU085_00431	HMPREF0351_10500	M7W_664
	Esp	*esp*	-	EFAU004_02750	EFAU085_02821	-	-
	Scm	*scm*	-	EFAU004_02838	EFAU085_02896	HMPREF0351_12673	M7W_2688
	SgrA	*sgrA*	-	EFAU004_01513	EFAU085_01549	HMPREF0351_11523	-
Antiphagocytosis	Capsule	*cpsA/uppS*	orf01754	EFAU004_01724	EFAU085_01748	HMPREF0351_11682	M7W_1191
		cpsB/cdsA	orf01753	EFAU004_01723	EFAU085_01747	HMPREF0351_11681	M7W_1192
		cpsC	orf02192	-	-	-	-
		cpsD	-	-	-	-	-
		cpsE	-	-	-	-	-
		cpsF	-	-	-	-	-
		cpsG	-	-	-	-	-
		cpsH	-	-	-	-	-
		cpsI	-	-	-	-	-
		cpsJ	-	-	-	-	-

		cpsK	orf02190	-	-	-	-
	Capsule(Klebsiella)		orf01976	-	-	-	-
Biofilm formation	BopD	*bopD*	orf00234; orf02171	EFAU004_00405	EFAU085_00344	HMPREF0351_10415	M7W_580
	Fsr locus	*fsrA*	-	-	-	-	-
		fsrB	-	-	-	-	-
		fsrC	-	-	-	-	-
Enzyme	Gelatinase	*gelE*	-	-	-	-	-
	Hyaluronidase	Undetermined	-	-	-	-	-
		Undetermined	-	-	-	-	-
	SprE	*sprE*	-	-	-	-	-
Toxin	Cytolysin	*cylA*	-	-	-	-	-
		cylB	-	-	-	-	-
		cylI	-	-	-	-	-
		cylL-l	-	-	-	-	-
		cylL-s	-	-	-	-	-
		cylM	-	-	-	-	-
		cylR1	-	-	-	-	-
		cylR2	-	-	-	-	-
Immune evasion	Capsule(Streptococcus)	*cps4I*	orf02186	-	-	-	-

Supplementary Table S3. Putative plasmids identification against a gram-positive database using PlasmidFinder of *Enterococcus faecium* BGPAS1-3.

Database	Plasmid	Query / Template length	Contig	Position in contig	Note	Accession number
Rep3	rep29	738/738	NODE_57_length_4047_cov_64.355867	2822..3559	CDS1(pGL)	HQ696461
Inc18	rep2	1494/1494	NODE_53_length_5413_cov_25.055240	2093..3586	orf1(pRE25)	X92945
Rep3	rep18a	933/933	NODE_44_length_9440_cov_55.189627	1649..2581	repA(p200B)	AB158402
RepA_N	repUS15	1041/1041	NODE_26_length_43465_cov_12.337002	1875..2915	repA(pNB2354p1)	CP004064
Inc18	rep1	1417/1491	NODE_32_length_22852_cov_11.273223	15274..16690	repE(pAMbeta)	AF007787
Rep3	repUS42	763/1158	NODE_45_length_7550_cov_85.6793675	5222..5982	repB(pVF18)	JN172910
Rep2	repUS53	661/666	NODE_65_length_2410_cov_162.600526	1728..2381	repB(pJB01)	AY425961

Supplementary Table S4. Putative genes involved in the synthesis of essential amino acids in *Enterococcus faecium* BGPAS1-3.

Pathway Mapping (1686906669, BGPAS1-3)	Entry	Symbol	Name
00250 Alanine, aspartate and glutamate metabolism (2)	K00814	GPT, ALT	alanine transaminase [EC:2.6.1.2]
	K11540	CAD	carbamoyl-phosphate synthase/aspartate carbamoyltransferase/dihydroorotase [EC:6.3.5.5 2.1.3.2 3.5.2.3]
00270 Cysteine and methionine metabolism (1)	K00558	DNMT1, dcm	DNA (cytosine-5)-methyltransferase 1 [EC:2.1.1.37]
00290 Valine, leucine, and isoleucine biosynthesis (1)	K00835	avtA	valine--pyruvate aminotransferase [EC:2.6.1.66]
00220 Arginine biosynthesis (1)	K00814	GPT, ALT	alanine transaminase [EC:2.6.1.2]

Note: The number in parentheses $^{()}$ represents the number of genes based on KAAS analysis.
